# Supplementary material for: High-speed three-dimensional photoacoustic computed tomography for preclinical research and clinical translation
Source: Nat Commun. 2021 Feb 9;12:882. doi: 10.1038/s41467-021-21232-1 (PMC7873071; doi:10.1038/s41467-021-21232-1)
Supplement: Supplementary file 1 — Supplementary Information [file 41467_2021_21232_MOESM1_ESM.pdf]

Supplementary Information

High-Speed Three-Dimensional Photoacoustic Computed Tomography for Preclinical Research and Clinical Translation

Li Lin,† Peng Hu,† Xin Tong,† Shuai Na,† Rui Cao, Xiaoyun Yuan, David C. Garrett, Junhui Shi, Konstantin Maslov, and Lihong V. Wang*

†These authors contributed equally to this work.

*Corresponding author. Email: LVW@caltech.edu

Supplementary Figures

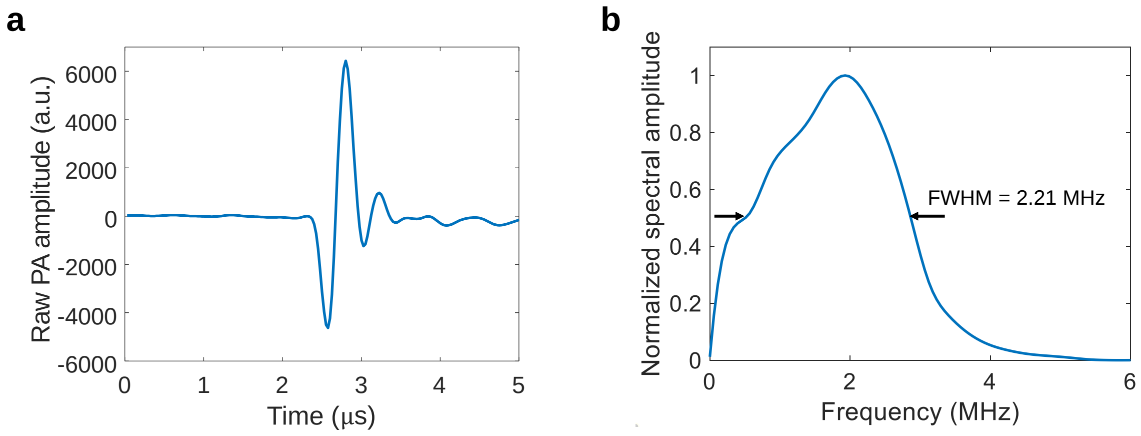

Supplementary Figure 1. Electrical impulse response of the 3D-PACT system. **a** The averaged radio frequency signal from all the ultrasonic transducer elements corresponding to a point PA source placed near the center of the imaging aperture. **b** Fourier transform amplitude of the radio frequency signal in (a), showing the bandwidth of the transducer array to be 2.21 MHz. The point source was created by fixing an aluminum particle (~50 µm) in an agar phantom. The particle was small enough to be regarded as a spatial point source for the 3D-PACT system. FWHM, full width at half maximum.

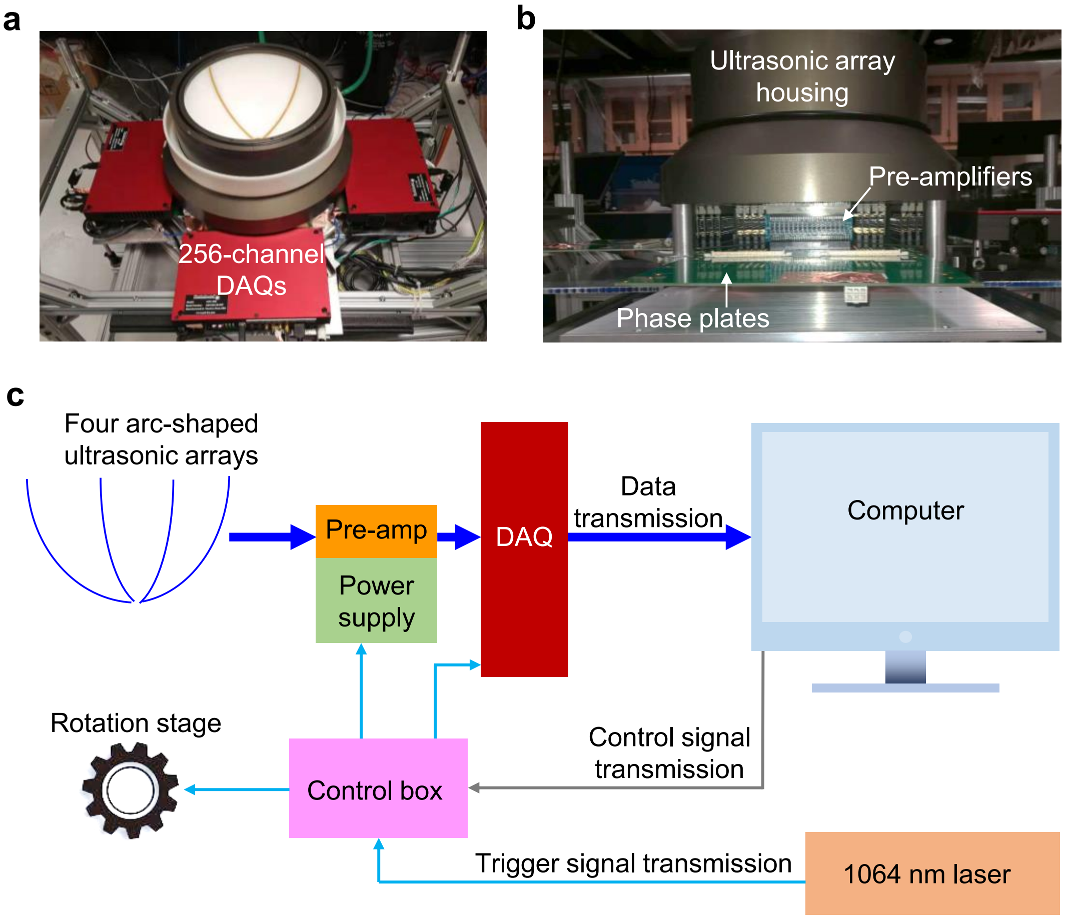

Supplementary Figure 2. Photographs and signal flow diagram of the 3D-PACT system. **a** Photograph of the system with the imaging platform removed. DAQ, data acquisition module. **b** Photograph of the system with the data acquisition modules removed, showing the pre-amplifier circuits and phase plates beneath the ultrasonic array housing. **c** Signal flow diagram of the system. The control box controlled the trigger signal transmission from the laser.

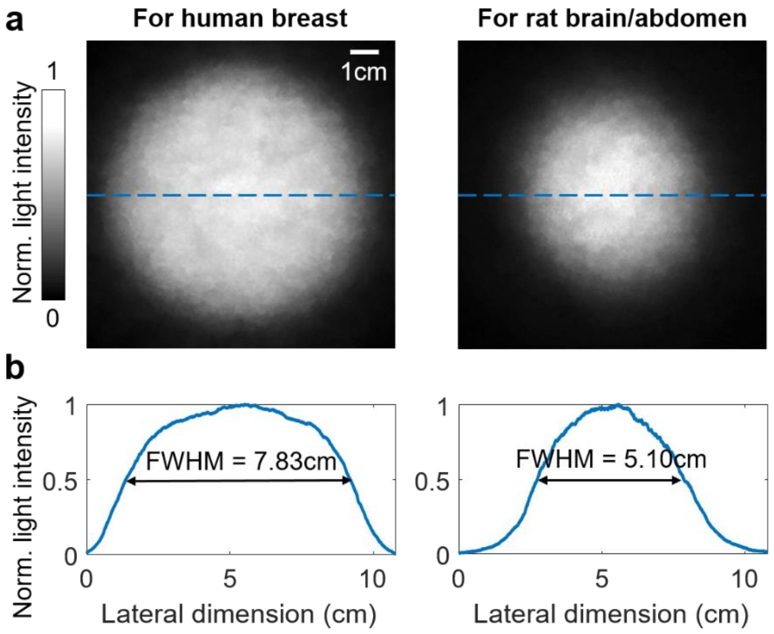

Supplementary Figure 3. a Normalized light intensity distribution measured near the tissue surface. **b** The light intensity distribution along the blue dashed lines in (a). The beam diameters, defined as the full width at half maximum (FWHM) of the intensity distribution, were 7.8 cm and 5.1 cm for human breast and rat brain/abdomen imaging, respectively.

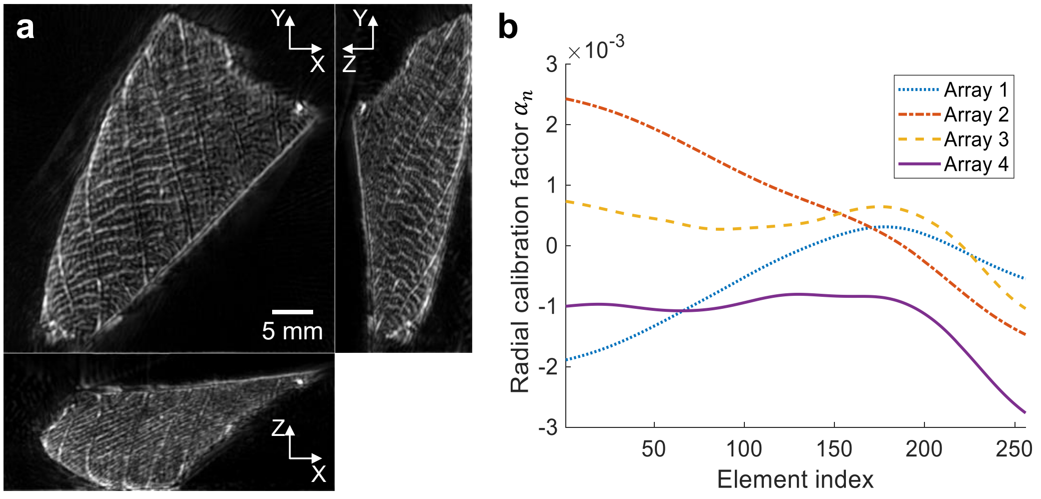

Supplementary Figure 4. a Maximum amplitude projection (MAP) images acquired before correcting the geometric defects of the ultrasonic arrays. The images reconstructed with defect correction are shown in Fig. 1d. **b** The ratio α_n of the geometric defect of each ultrasonic transducer element to the radius.

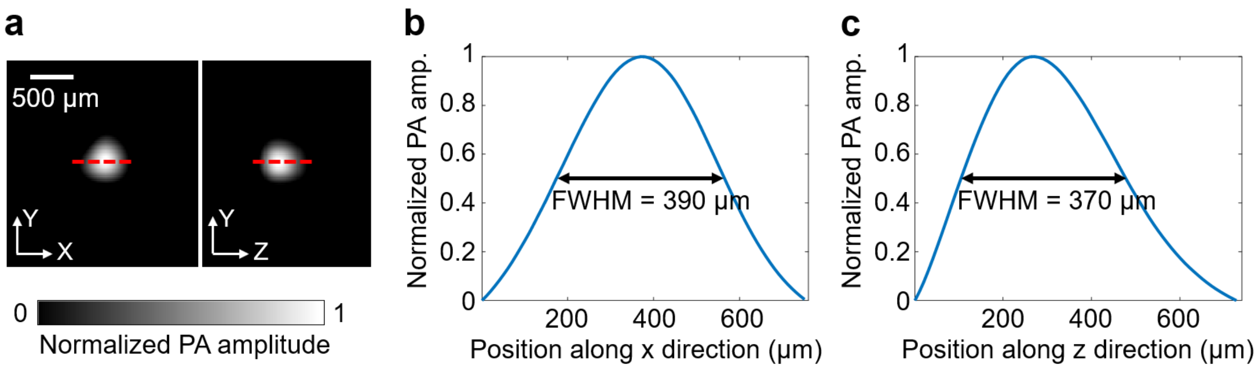

Supplementary Figure 5. Quantification of the spatial resolution of the 3D-PACT system. **a** MAP images of an aluminum particle (~50 μm) placed near the center of the imaging aperture. **b**, **c** The photoacoustic amplitude distribution along the red dashed lines in (a). The horizontal resolution, defined as the full width at half maximum (FWHM) of the amplitude distribution, is 390 μm; Similarly, the elevational resolution is 370 μm.

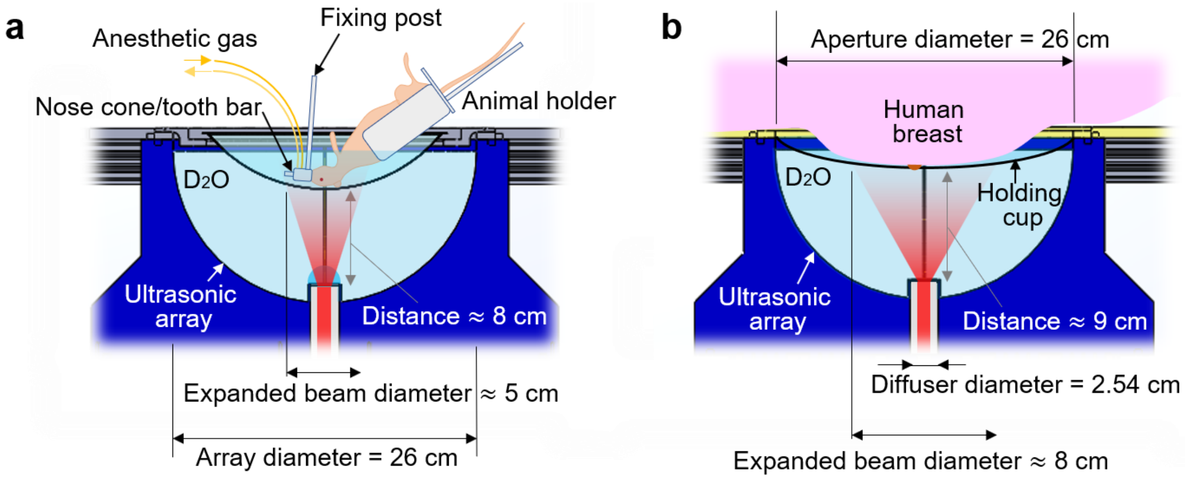

Supplementary Figure 6. a Setup of the rat brain imaging. The rat head was mounted reversely in a waterproof nose cone and the light was delivered to the rat cortex from the bottom. **b** Setup of the human breast imaging. The breast was slightly compressed against the chest wall by the holding cup, which was around 3 cm in depth. D_2O, deuterium oxide.

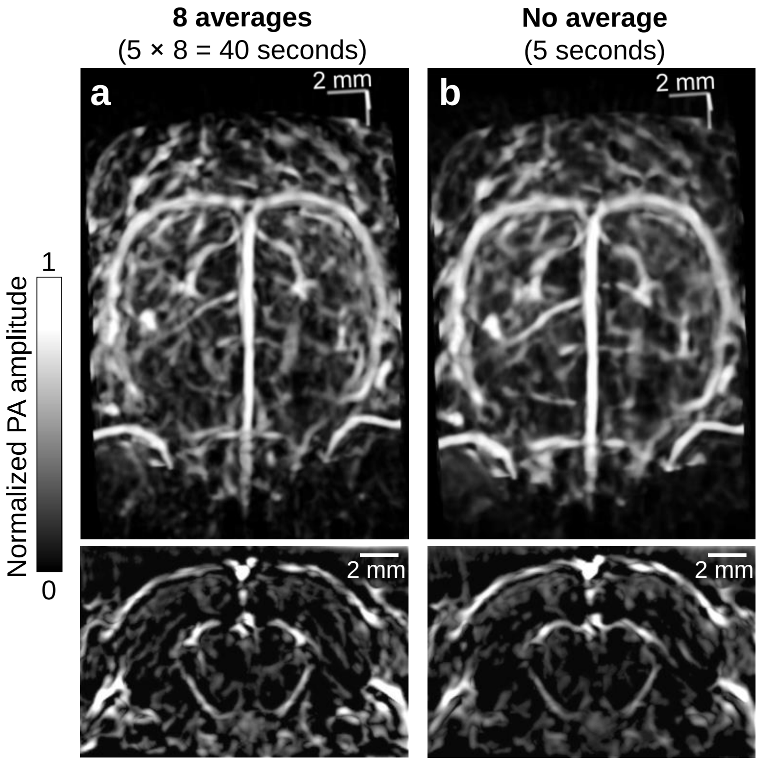

Supplementary Figure 7. Comparison of the rat brain images acquired with and without averaging. **a** Perspective and cross-sectional images acquired by averaging over eight 5-second scans. **b** Perspective and cross-sectional images acquired by single 5-second scan.

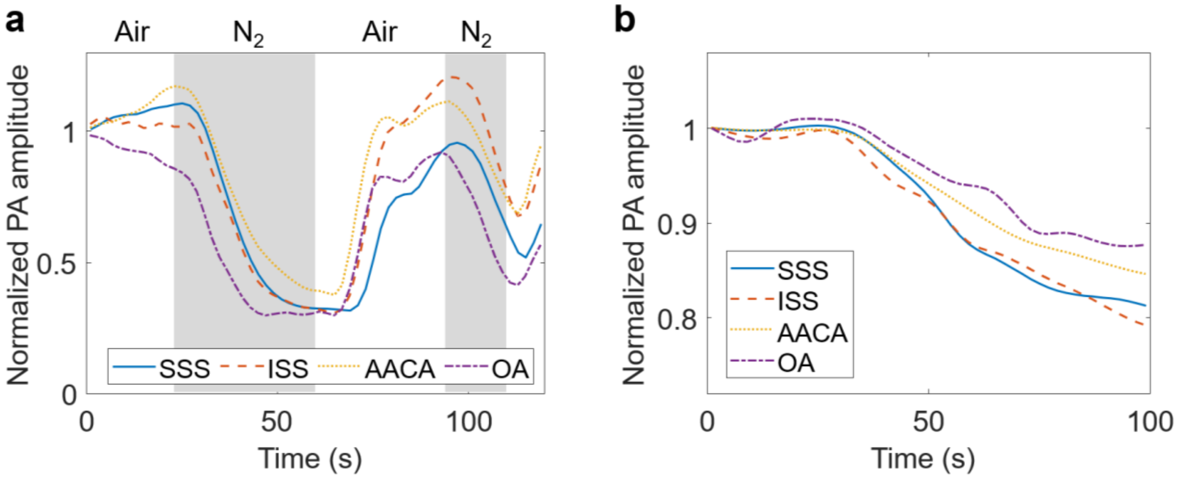

Supplementary Figure 8. PA signal variations in four major cerebral vessels measured by 3D-PACT. **a** PA signal changes during hypoxic challenges. **b** PA signal changes from deep to light anesthesia. AACA, azygos of the anterior cerebral artery; ISS, inferior sagittal sinus; OA, olfactory artery; SSS, superior sagittal sinus.

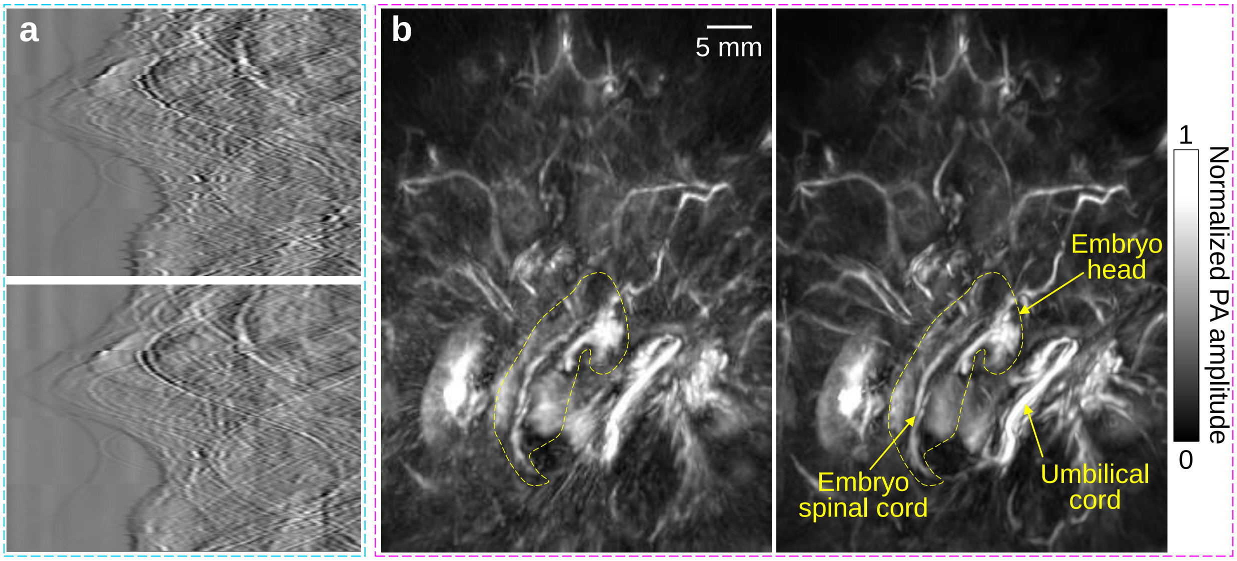

Supplementary Figure 9. Imaging of a pregnant rat abdomen with time-gated motion correction. **a** PA signals before (top) and after (bottom) correcting respiratory motion. **b** MAP images reconstructed from the PA signals before (left) and after (right) motion correction.

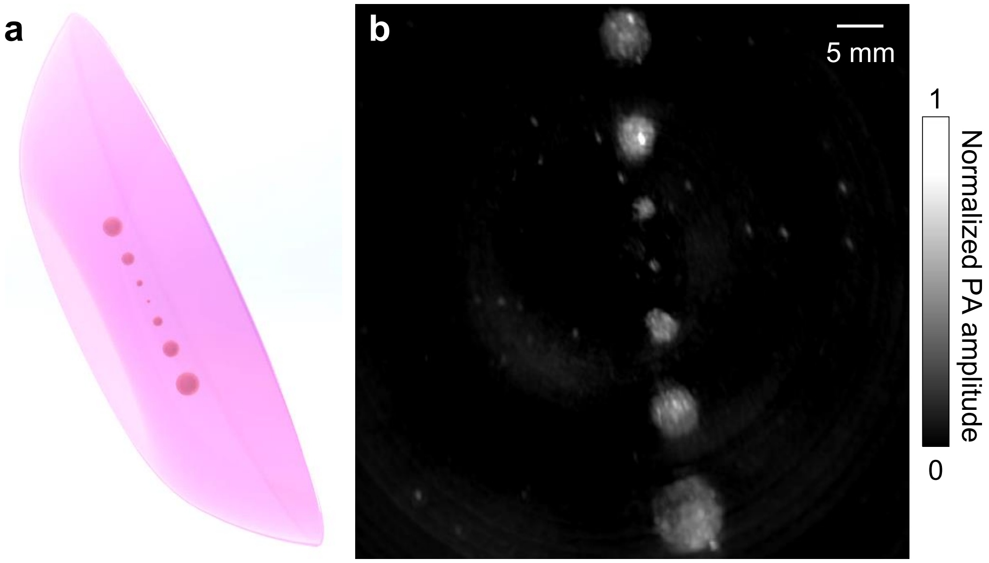

Supplementary Figure 10. Imaging of tumor phantoms with different sizes embedded in a breast-mimicking phantom. **a** Sketch of the breast-mimicking phantom, which consisted of clear agarose (3%), black acrylic ink (0.0125%), and 20% intralipid (3.6%)[1]. Seven tumor phantoms were embedded at a depth of 2 cm. The diameters of the tumors were 1 mm, 1.5 mm, 2 mm, 2.5 mm, 3 mm, and 3.5 mm respectively. The absorption coefficients μ_a of the tumors and breast phantom

were 0.105 cm^{-1} and 0.05 cm^{-1}, respectively[2,3]. The reduced scattering coefficient μ_s' of the phantoms was 5 cm^{-1} at 1064 nm[3,4]. **b** MAP image of the breast phantom acquired by 3D-PACT, which revealed the smallest tumor phantom (1-mm diameter). The bright dots in the image background were caused by air bubbles embedded in the phantom.

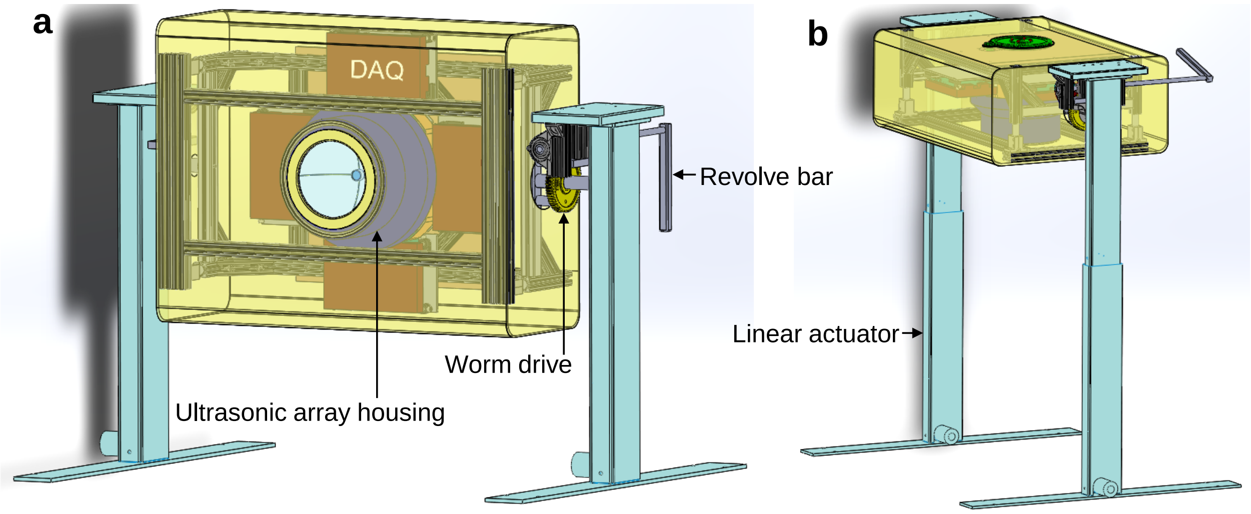

Supplementary Figure 11. Potential configurations of the 3D-PACT system with two examples of 90-degree (a) and 180-degree rotation (b) to fit different imaging positions and requirements.

Supplementary Methods

Pseudo-Code and Description of the Universal Back-Projection Algorithm

1. Spatial interpolation

Numerically double the number of scan angles and the number of elements by applying spatial interpolation to detected signals[5].

2. Virtual array generation

Allocate memory space for a virtual array consisting of all locations and normal directions of elements in a 3D scan.

Build a Cartesian coordinate for the initial four-arc array.

Calculate locations and normal directions of all elements in the array based on specifications in array fabrication.

```
for (nScan = 0 to all scan angles) {
    Calculate element locations and normal directions for the nScan-th angle based on rigid transformation.
    Add the calculated locations and directions to the virtual array.
}
```

Apply radial correction to all elements in the virtual array (using radial correction factors obtained from point source experiments).

3. Image reconstruction

Initialize all voxels in the to-be-reconstructed image as zero.

```
for (n = 0 to all elements in the virtual array) {
    for (m = 0 to all voxels in the image) {
        Calculate distance between the m-th voxel and the n-th element.
        Calculate the backprojection term[6].
        Calculate the backprojection weight.
        Add the weighted backprojection term to the m-th voxel.
    }
}
```

Supplementary References

1. Staveren, H.J., Moes, C.J.M., Marie, J., Prahl, S.A. & Gemert, M.J.C. Light scattering in intralipid-10% in the wavelength range of 400–1000 nm. *Appl. Opt.* **30**(31), 4507–4514 (1991).
2. Grosenick, D., Rinneberg, H., Cubeddu, R. &Taroni, P. Review of optical breast imaging and spectroscopy. *J. Biomed. Opt.* **21**(9), 091311 (2016).
3. Durduran, T., *et al*. Bulk optical properties of healthy female breast tissue. *Phys. Med. Biol.* **47**, 2847–2861 (2002).
4. Generic tissue optical properties. *https://omlc.org/news/feb15/generic_optics/index.html*
5. Hu, P., Li, L., Lin, L. & Wang, L.V. Spatiotemporal antialiasing in photoacoustic computed tomography. *IEEE Trans. Med. Imaging* **39**(11), 3535–3547 (2020).
6. Xu, M. & Wang, L.V. Universal back-projection algorithm for photoacoustic computed tomography. *Phys. Rev. E* **71**(1), 016706 (2005).
