# Supplementary material for: High-speed three-dimensional photoacoustic computed tomography for preclinical research and clinical translation
Source: Nat Commun. 2021 Feb 9;12:882. doi: 10.1038/s41467-021-21232-1 (PMC7873071; doi:10.1038/s41467-021-21232-1)
Supplement: Supplementary file 3 — Description of Additional Supplementary Files [file 41467_2021_21232_MOESM3_ESM.pdf]

Description of Additional Supplementary Files

Supplementary Movie 1. Volumetric image of a rat brain *in vivo*, showing detailed angiographic structures from the cortex to the Circle of Willis. Scale bars: 2 mm.

Supplementary Movie 2. Volumetric images of the rat brain: (I) intrinsic functional connectivity across spatially separated brain regions; (II) cortical hemodynamics in response to electrical stimulation to the front limbs.

Supplementary Movie 3. Volumetric image of the right human breast (cup 36C) *in vivo*, revealing blood vessels as deep as 4 cm. The image was acquired within a single breath hold of 10 seconds. Scale bars: 5 mm.

Supplementary Movie 4. Volumetric image of the left human breast *in vivo*. Scale bars: 5 mm.
