# Supplementary material for: High-speed three-dimensional photoacoustic computed tomography for preclinical research and clinical translation
Source: Nat Commun. 2021 Feb 9;12:882. doi: 10.1038/s41467-021-21232-1 (PMC7873071; doi:10.1038/s41467-021-21232-1)
Supplement: Supplementary file 8 — Reporting Summary [file 41467_2021_21232_MOESM8_ESM.pdf]

nature research

Corresponding author(s): Lihong V. Wang

Last updated by author(s): Dec 1, 2020

Reporting Summary

Nature Research wishes to improve the reproducibility of the work that we publish. This form provides structure for consistency and transparency in reporting. For further information on Nature Research policies, see our Editorial Policies and the Editorial Policy Checklist.

Statistics

For all statistical analyses, confirm that the following items are present in the figure legend, table legend, main text, or Methods section.

n/a	Confirmed	
☒	☐	The exact sample size (n) for each experimental group/condition, given as a discrete number and unit of measurement
☒	☐	A statement on whether measurements were taken from distinct samples or whether the same sample was measured repeatedly
☒	☐	The statistical test(s) used AND whether they are one- or two-sided<br>*Only common tests should be described solely by name; describe more complex techniques in the Methods section.*
☒	☐	A description of all covariates tested
☒	☐	A description of any assumptions or corrections, such as tests of normality and adjustment for multiple comparisons
☒	☐	A full description of the statistical parameters including central tendency (e.g. means) or other basic estimates (e.g. regression coefficient) AND variation (e.g. standard deviation) or associated estimates of uncertainty (e.g. confidence intervals)
☒	☐	For null hypothesis testing, the test statistic (e.g. F, t, r) with confidence intervals, effect sizes, degrees of freedom and P value noted<br>*Give P values as exact values whenever suitable.*
☒	☐	For Bayesian analysis, information on the choice of priors and Markov chain Monte Carlo settings
☒	☐	For hierarchical and complex designs, identification of the appropriate level for tests and full reporting of outcomes
☒	☐	Estimates of effect sizes (e.g. Cohen's d, Pearson's r), indicating how they were calculated

Our web collection on statistics for biologists contains articles on many of the points above.

Software and code

Policy information about availability of computer code

Data collection	The data acquisition system was operated using NI LabVIEW 2018 (64-bit). The imaging system was controlled by a graphical user interface (GUI) generated by MATLAB 2018.
Data analysis	The reconstruction algorithm and data processing methods are described in detail in the Methods. The image reconstruction (universal back-projection algorithm, cited in the manuscript) was implemented in C++ with GPU acceleration. The image processing codes are uploaded with the experimental data. Supplementary Movie 1 was generated using VolView 3.4; Supplementary Movie 2 was made using a specialized 3D visualization software package, namely 3D Photoacoustic Visualization Studio (cited in the manuscript); Supplementary Movies 3 and 4 were produced using the visvis library in Python 3.7.4 (code uploaded with the data).

For manuscripts utilizing custom algorithms or software that are central to the research but not yet described in published literature, software must be made available to editors and reviewers. We strongly encourage code deposition in a community repository (e.g. GitHub). See the Nature Research guidelines for submitting code & software for further information.

Data

Policy information about availability of data

All manuscripts must include a data availability statement. This statement should provide the following information, where applicable:

- Accession codes, unique identifiers, or web links for publicly available datasets
- A list of figures that have associated raw data
- A description of any restrictions on data availability

All data are available within the Article and Supplementary Files, or available from the corresponding author upon reasonable request. Imaging data from in vivo experiments and study approvals issued by the IACUC and IRB are available in the online folder: https://figshare.com/articles/dataset/3D-PACT_Data_and_Codes/13114544

Field-specific reporting

Please select the one below that is the best fit for your research. If you are not sure, read the appropriate sections before making your selection.

- [x] Life sciences
- [ ] Behavioural & social sciences
- [ ] Ecological, evolutionary & environmental sciences

For a reference copy of the document with all sections, see nature.com/documents/nr-reporting-summary-flat.pdf

Life sciences study design

All studies must disclose on these points even when the disclosure is negative.

Sample size	We imaged three rats and one healthy human subject. No sample size calculation was performed. The number of rats and human subject was chosen to sufficiently demonstrate our imaging technology and application.
Data exclusions	No imaging data was excluded. In the manuscript, we show the rat brain/abdomen images with best qualities and the images of both breasts. The image quality is slightly affected by the placement (i.e., position and orientation) of the object.
Replication	Similar imaging results have been obtained over multiple measurements with reported sample sizes. The sample was imaged multiple times during each experiment (every 1-2 weeks). All replication attempts were successful.
Randomization	The experiments were not randomized. Randomization was not relevant to this study because we are not comparing between samples.
Blinding	Blinding was not applicable during experiments and outcome assessment because we do not compare between samples or populations. Data analyses were carried out in angiographic images with high signal-to-background ratios.

Reporting for specific materials, systems and methods

We require information from authors about some types of materials, experimental systems and methods used in many studies. Here, indicate whether each material, system or method listed is relevant to your study. If you are not sure if a list item applies to your research, read the appropriate section before selecting a response.

Materials & experimental systems

n/a	Involved in the study
x	Antibodies
x	Eukaryotic cell lines
x	Palaeontology and archaeology
	x Animals and other organisms
	x Human research participants
x	Clinical data
x	Dual use research of concern

Methods

n/a	Involved in the study
x	ChIP-seq
x	Flow cytometry
x	MRI-based neuroimaging

Animals and other organisms

Policy information about studies involving animals; ARRIVE guidelines recommended for reporting animal research

Laboratory animals	One-month-old rats (Hsd:Sprague Dawley SD, Harlan Co.; 100-120g body weight, male) were used for functional brain imaging. A three-month-old pregnant rat (Hsd:Sprague Dawley SD, Harlan Co.; Timed mated, 20 days) was used for abdomen imaging.
Wild animals	No wild animals were used in this study.
Field-collected samples	No field collected samples were used in this study.
Ethics oversight	All the animal experiments followed a protocol approved by the Institutional Animal Care and Use Committee (IACUC) of the California Institute of Technology (Protocol number: 20-1737).

Note that full information on the approval of the study protocol must also be provided in the manuscript.

Human research participants

Policy information about studies involving human research participants

Population characteristics	In the human imaging protocol, we included healthy female subjects of all races and ethnic groups. The subject we recruited in this study was a 31-year-old healthy female Asian volunteer with 36C-cup breasts.

Recruitment	There was no self-selection. The imaging subject (not Caltech personnel) knew of our study by visiting our lab.
Ethics oversight	The human breast imaging followed a protocol approved by the Institutional Review Board (IRB) of the California Institute of Technology (Protocol number: 20-0985). IRB and IACUC were aware of both protocols before approving.

Note that full information on the approval of the study protocol must also be provided in the manuscript.
